# The Significance of Coherent Transformation on Grain Refinement and Consequent Enhancement in Toughness

**DOI:** 10.3390/ma13225095

**Published:** 2020-11-12

**Authors:** Xiucheng Li, Jingxiao Zhao, Lili Dong, R. Devesh Kumar Misra, Xuemin Wang, Xuelin Wang, Chengjia Shang

**Affiliations:** 1 Collaborative Innovation Center of Steel Technology, University of Science and Technology Beijing, 30 Xueyuan Road, Haidian District, Beijing 100083, China; xiuchengli@ustb.edu.cn (X.L.); zhjingxiao@yeah.net (J.Z.); 18911237610@163.com (L.D.); wxm@mater.ustb.edu.cn (X.W.); xuelin2076@163.com (X.W.); 2 Department of Metallurgical, Materials and Biomedical Engineering, University of Texas at El Paso, 500 W. University Avenue, El Paso, TX 79968, USA

**Keywords:** bainitic steel, coherent transformation, toughness, ductile-to-brittle transition, misorientation

## Abstract

Coherent transformation is considered to be an effective approach to refine the microstructure and enhance toughness of structural steels. However, there are gaps in the knowledge on the key aspects of microstructure that govern toughness. In this regard, a low alloyed experimental steel with lean chemistry was subjected to a simple heat treatment involving austenitization at different temperatures, followed by quenching and tempering to obtain bainitic microstructures with different boundary composition. The microstructure of the four experimental steels was characterized by electron backscattered diffraction and mechanical properties were determined. The study indicated that the density of high angle grain boundaries does not adequately reflect the change of ductile-to-brittle transition temperatures (DBTT) of the experimental steels. Thus, we propose here a new mechanism on reducing DBTT from the perspective of misorientation of boundary, which takes into consideration these aspects in defining DBTT. One is inhibition effect on cleavage fracture by boundaries with high {100}-plane misorientation angles, and the other is ductility improvement by boundaries with high {110}-plane misorientation angles. Furthermore, the contribution of prior austenite grain boundary, packet boundary, block boundary, and sub-block boundary on toughness is also analyzed.

## 1. Introduction

**Figure 1:**
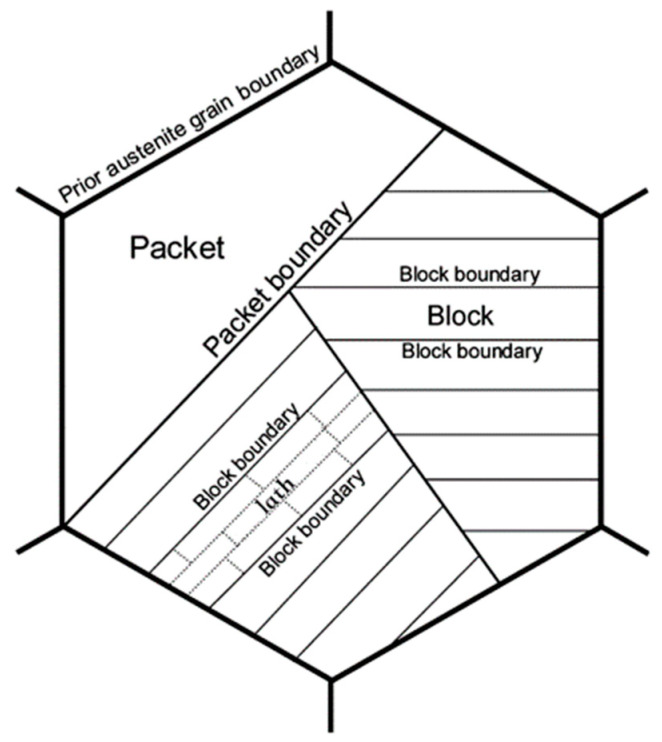
Schematic representation of microstructural hierarchy of lath bainite/martensite based on the available knowledge in literature [6].

Grain refinement is considered as the most effective approach to simultaneously increase strength and toughness of metallic materials at the same time. Hall–Petch and Cottrell–Petch relationships are widely accepted in the development of steels, which indicate that the yield strength is inversely proportional to the square root of the grain diameter, while the ductile-to-brittle transition temperature (DBTT) is inversely proportional to the half power of the grain diameter [1,2]. The concept of grain refinement is clear for ferritic/pearlitic steels [3,4,5] because they have relatively simple crystallographic structure. However, for bainite and martensite microstructures formed by coherent transformation, there are multi-levels of crystallographic structures. Figure 1 [6] schematically illustrates the typical lath bainite/martensite structure with three-level hierarchy within one prior austenite grain (PAG): martensite lath, block, and packet. Therefore, the concept of grain refinement for bainite/martensite steels is associated with the refinement of PAG, packet, block, or lath. Previous studies attribute the effective grain size that affects the toughness and DBTT to PAG [7,8], packet [9,10,11,12], or block and lath [13,14,15].

The relationship between the effective grain size on toughness of bainite/martensite steels remains unclear. A number of studies suggested that the high angle grain boundaries (HAGBs) can enhance toughness by decreasing the mean free path of a cleavage crack, i.e., forcing the crack to deviate. Thus, the concept of misorientation angle (θ) was proposed to evaluate the effect of different boundaries on toughness. Theoretically, the higher is the misorientation angle, the more significant is the toughening effect of the boundary. However, there is no unified definition of HAGB until now. Some studies considered HAGBs as interfaces with misorientation angles greater than 15° [16,17], while others suggest the threshold value of misorientation angle to be 45° [18,19,20]. More recently, it was illustrated that DBTT does not change with the density of HAGBs in a regular manner [12,21], which leads us to think even deeper as to how to evaluate the effective grain size of bainite/martensite steels. Based on the aforementioned background, the objective of the present study was to identify, classify, and quantify the boundaries of experimental steels and utilize the misorientation angles of specific crystallographic planes together with overall misorientation angle to describe boundaries. The ultimate aim was to reveal the contributions of different types of boundaries on toughness.

## 2. Experimental Procedure

**Figure 2:**
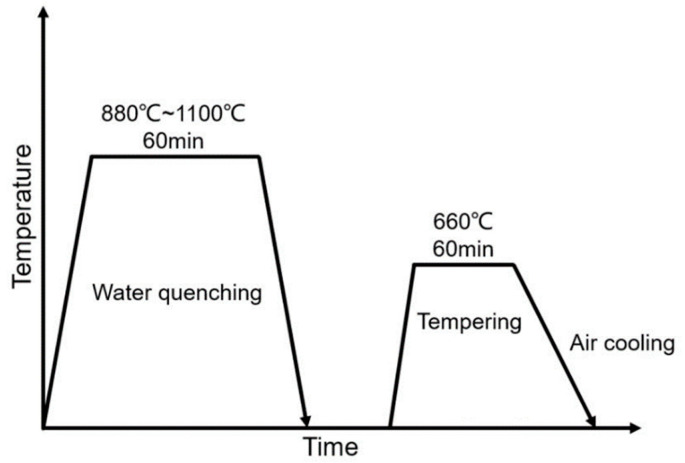
Schematic representation of processing schedule of experimental steels.

The chemical composition of the experimental steel in weight percent was 0.09% C, 0.26% Si, 1.05% Mn, 2.31% Cr + Ni + Mo, 0.08% Nb + V + Ti, 0.006% P, 0.0017% S, and balance Fe. Steel blanks were cut from a 25 mm thick plate and heated to temperatures of 880, 930, 1050, and 1100 °C, respectively, held for 60 min, water quenched, followed by tempering at 660 °C for 60 min. The processing scheme is shown in Figure 2. The samples corresponding to different austenitization temperatures are referred henceforth as A880, A930, A1050, and A1100. The specimens for tensile test and Charpy V-notch impact test were prepared from the mid-thickness of the plate. Dog bone bar-shaped (effective tested zone: Φ10 mm × 50 mm, two specimens for each sample) tensile specimens were tested at room temperature and a strain rate of 1.0 × 10^−3^/s. Standard Charpy V-notch impact specimens (10 mm × 10 mm × 55 mm, three specimens for each sample and for each test temperature) were tested from room temperature to very low temperature (from −20 to −140 °C) to obtain a clear low shelf of impact absorbed energy for each sample. Scanning electron microscopy (SEM) equipped with electron backscattered diffraction (EBSD) was used for microstructure characterization. Step size was set at 0.2 μm, which is smaller than the variant size of all the samples. The EBSD data were interpreted using the HKL technology Channel 5 software. A self-written Python script was also employed to compute misorientation angles, {100}-plane specific misorientation angles ({100}-SMA) and {110}-plane specific misorientation angles ({110}-SMA). The misorientation matrix between the two orientations was calculated by:

T = M_1_·M_2_^−1^ (1)

where T is the misorientation matrix and M_1_ and M_2_ are the orientation matrices of two different orientations, respectively.

The misorientation angle of two different orientations was calculated by:

(2) $$\;\theta =cos^{-1}\left(\frac{T_{11}+T_{22}+T_{33}}{2}\right)$$

In body centered cubic structure, there are 9 different {100}-SMAs and 36 different {110}-SMAs because of symmetry. Since the mechanical behavior, such as the propagation of cleavage crack or crystal slip, tends to occur preferentially in the case of lowest resistance, the lowest value of {100}-SMAs or {110}-SMAs was selected as the appropriate value [21]. Another self-written computer program was used for prior austenite reconstruction and to identify the variants boundary.

## 3. Results and Discussion

**Figure 3:**
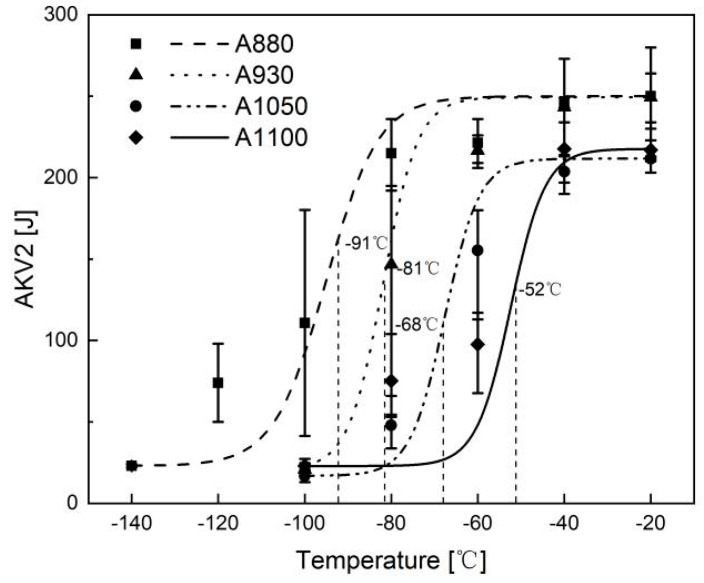
Impact absorbed energy as a function of temperature for different austenitizing temperatures.

**Figure 4:**
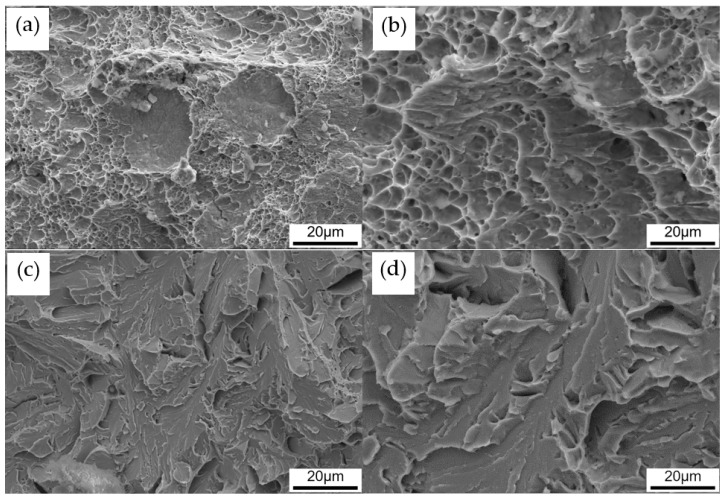
SEM morphology showing the fracture surface near the crack-initiation site of Charpy impact specimens of: A880 (**a**,**b**); and A1100 (**c**,**d**). Both specimens failed at −100 °C.

The tensile properties and DBTT are listed in Table 1. As the austenitization temperature was increased, yield strength, tensile strength, and total elongation decreased. This means that lower austenitization temperature increases strength and ductility at the same time. DBTT of each sample was estimated as the temperature which shows intermediate absorption energy of upper and lower shelf, as shown in Figure 3. As expected, DBTT decreased with the decrease of austenitization temperature, because toughness is reflective of ductility. Figure 4 shows the fractographs of Charpy impact specimens with typical ductile fracture and brittle fracture. Figure 4a is the fractograph of A880 tested at −100 °C. Although the test temperature is low, there are a number of dimples, as shown in Figure 4b. It indicates significant plastic deformation occurred during fracture. Figure 4c,d shows the fractograph of A1100 Charpy impact specimen tested at −100 °C. It is a typical cleavage fracture, once the crack formed, it propagated quickly and almost no plastic deformation occurred during the fracture. Since the experimental steel has a lean chemical composition design, it is reasonable to conclude that the change in mechanical properties is related to the difference in boundary types and corresponding densities.

**Figure 5:**
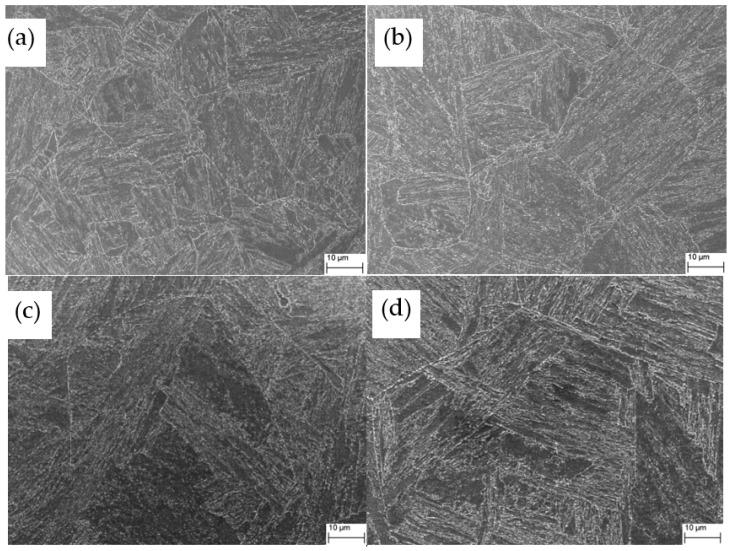
SEM micrographs of samples with different austenitizing temperatures: (**a**) A880; (**b**) A930; (**c**) A1050; and (**d**) A1100.

**Figure 6:**
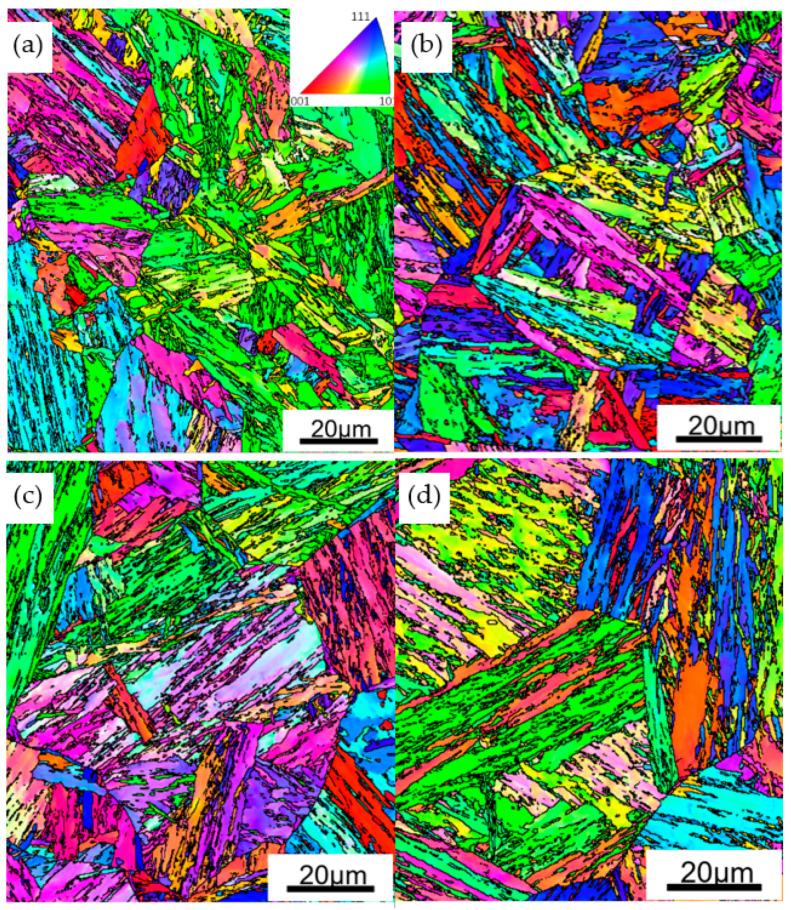
Inverse pole figure maps and boundary distribution (black lines: θ > 5°) as obtained by EBSD of: (**a**) A880; (**b**) A930; (**c**) A1050; and (**d**) A1100.

Traditional methods for microstructure observation, such as light optical microscopy or SEM, may show the boundaries, but these observations can hardly distinguish the different types of boundaries in bainitic/martensitic microstructures because the boundaries do not have good discrimination in morphology. Figure 5 shows the microstructure of samples with different austenitization temperatures as observed by SEM. The PAG and lath bundles can be distinguished to some extent, and there are some very fine carbides and M-A (martensite-retained austenite) formed in the microstructure. Large or necklace-like M-A that is detrimental to toughness was not observed. EBSD provides an approach to realize detailed characterizations of crystallographic structure in a large field of view [22,23,24]. Thus, our focus was on EBSD analysis. For EBSD studies, the FCC and BCC crystallographic structure was set and very few FCC grains were found, which implied that the influence of retained austenite on toughness can be ignored in the samples. Figure 6 shows the inverse pole figure (IPF) color maps obtained by processing the EBSD measurements. Here, different colors represent bainitic crystallographic units of bainite/martensite that agree with the orientation perpendicular to the observed plane, as indicated by the stereographic triangle in the inset. Using the data of Euler angles, the misorientation angles of boundaries were calculated, and the boundaries with misorientation angles greater than 5° are also delineated in Figure 6. The profile of PAG can be barely recognized; for A880 and A930, there are several PAGs in each EBSD map because the PAGs are competitively small. However, for A1050 and A1100, the PAG are much larger; thus, the EBSD map contains fewer PAGs. There are also more boundaries within the PAG, which are mainly variant boundaries including packet boundaries, block boundaries, and sub-block boundaries. To make a comparison of different types of boundaries, a normalization was adopted by counting total length of each type of boundaries within same observation area from EBSD measurements.

**Figure 7:**
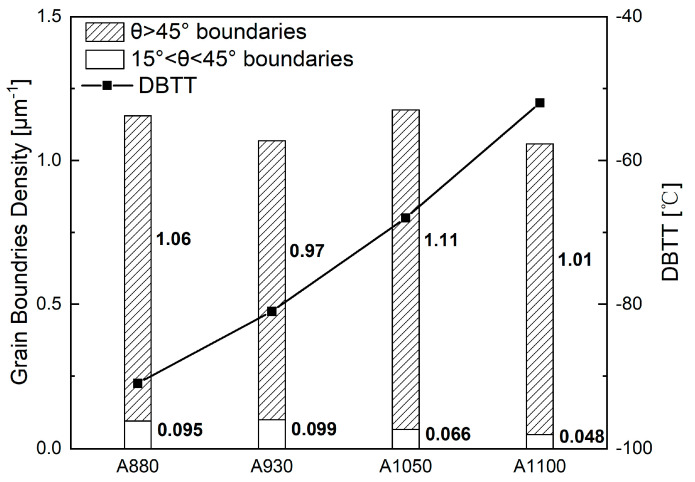
Grain boundary density (15° < θ < 45° and θ > 45°) and DBTT of experimental steels.

**Figure 8:**
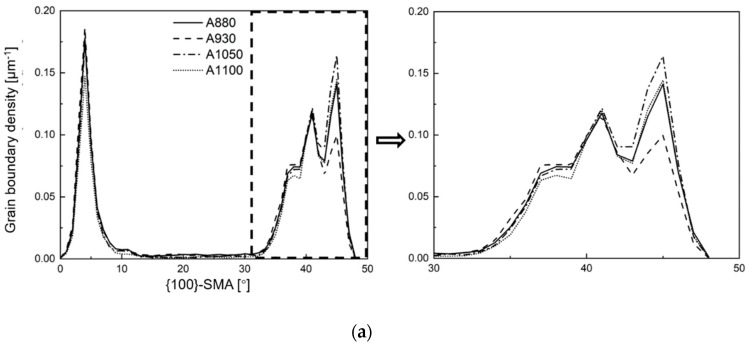

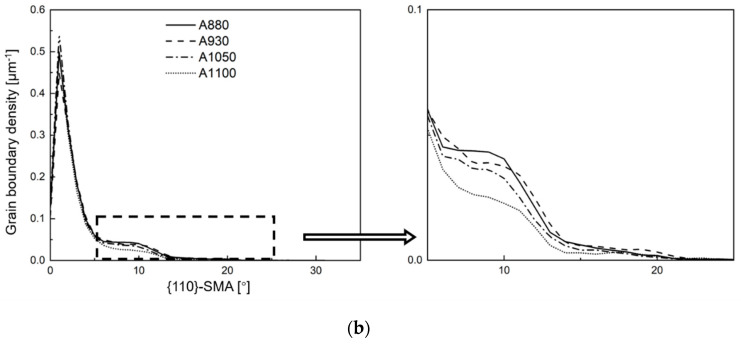
Boundary density distribution counted with: (**a**) {100}-SMA; and (**b**) {110}-SMA.

Figure 7 shows the quantitative results of boundaries according to their misorientation angles. The two frequently used threshold values 15° and 45° were selected to define HAGB. There is no significant difference in density of boundaries between the different samples. Comparatively, A1050 had the highest density of HAGBs (θ > 45°), and A930 had the lowest density in the four samples. A880 and A1050 also had higher density of HAGBs (θ > 15°). While some studies suggested that toughness can be improved by high density of HAGBs, our results do not follow the rule. In reality, misorientation angle is an overly simplistic criterion because, in crystals, the crystallographic plane and the crystallographic direction are the key factors that affect the mechanical properties. Overall, misorientation angle cannot adequately reflect information of misorientations of specific crystallographic plane/direction. As Morris et al. [25] and Ghosh et al. [26] pointed out in their studies, {100}-SMA is more relevant than the overall misorientation angle, when defining the effective grain size for cleavage fracture. Recent studies show that misorientation angle of {110} crystallographic plane can also influence toughness by increasing ductility [21]. Thus, in this study, the {100}-SMAs and {110}-SMAs of all boundaries (θ > 5°) were calculated and the distribution of corresponding boundary density is shown in Figure 8. Figure 8a shows boundary density counted with {100}-SMA. A1050 has relatively slightly higher boundary density when {100}-SMA is greater than 40° and A930 has lower boundary density in this range. More significant difference was observed in the boundary density distribution with {110}-SMA, which is shown in Figure 8b. While the four curves are very close to each other, considering that the boundaries with high {110}-SMAs play a more important role with regard to dislocation pile-up, the segment of curves above 5° was enlarged. It may be noted that there are also some differences. A880 has higher boundary density, followed by A930 and A1050, and the boundary density of A1100 above 7° is only about 2/3 of A880. Thus, it is not surprising that A880 had higher strength and ductility. Therefore, DBTT may be comprehensively influenced by two aspects: boundaries with high {100}-SMAs inhibit cleavage fracture and boundaries with high {110}-SMAs improve ductility. It is easy to understand that boundaries with high {100}-SMAs can improve toughness and lower DBTT [25,26,27]. The mechanism of high {110}-SMA boundary reducing DBTT is likely to reduce the length of dislocation pile-up [28,29] and influence the competitive relationship between crack extension and the operating dislocation source [30].

The most significant feature of coherent transformation is that the parent phase and the product phase have a fixed orientation relationship [27,31]; consequently, the overall misorientation and specific misorientation of the newly formed interfaces (variant boundaries) are not randomly distributed. This is a critical and important aspect to reveal the effect of coherent transformation on toughness. The microstructure of the four experimental steels consisted of lath bainite, which normally have Kurdjumov–Sachs (K-S) orientation relationship with the parent phase [22]. For K-S orientation relationship, there are 24 possible of variants within one PAG, which are referred to as V1–V24. Table 2 shows the characteristics of crystallographic orientation and classification methods for all the variants under K-S relationship [6,28]. According to four different close packed plane selection during transformation, 24 variants can be divided into four CP groups; each CP Group includes six variants. For example, V1–V6 belong to Group (CP1), and, among these six variants, block boundaries can be formed by V1–V2, V1–V3, V1–V5, and V1–V6 variant pairs and sub-block boundary is formed by V1–V4 variant pair. However, there is another way to divide the variants by Bain groups, which is, when the face centered cubic crystal changes to the body centered cubic crystal, the Bain strain will cause compression. This compression can be carried out along three mutually orthogonal axes, and the variants compressed along the same axis belong to one Bain group, thus three Bain groups are formed. According to Bain group, the overall misorientation information of different variant pairs can be effectively reflected [32]. Theoretically, the minimum misorientation angle formed by the variants from different Bain groups is ~47°, while the variation from the same Bain group can only form a maximum misorientation angle ~21°. Strictly speaking, the boundaries formed by variants form different CP groups and at the same time from different Bain groups need to be classified as packet boundaries and not block boundaries; for the same reason, sub-block boundary only has one probability of variant adjacency, which is V1–V4. Different types of boundaries contribute to strength, ductility, and toughness differently; even for one type of boundary, the contribution can be different.

**Figure 9:**
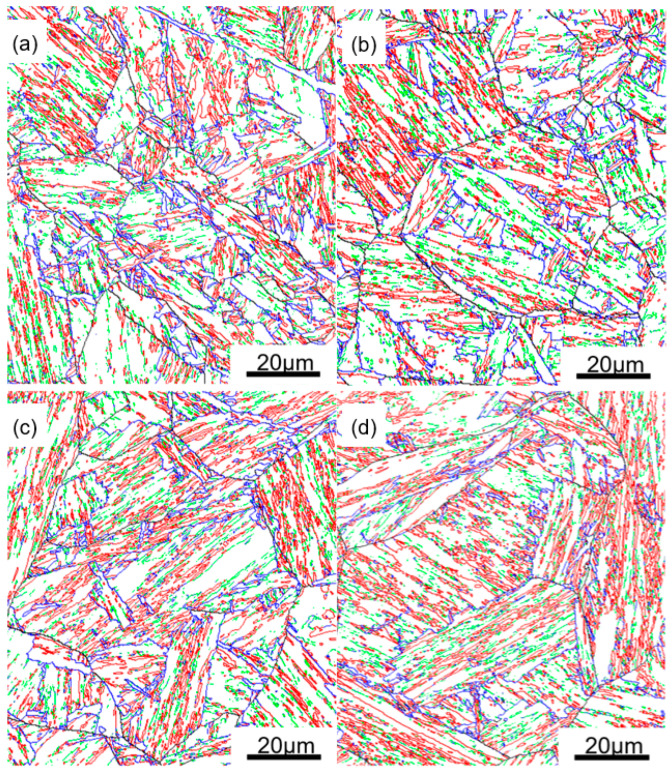
Different types of boundaries (black lines: PAGBs, blue lines: packet boundaries, red lines: block boundaries, green lines: sub-block boundaries) of: (**a**) A880; (**b**) A930; (**c**) A1050; and (**d**) A1100.

**Figure 10:**
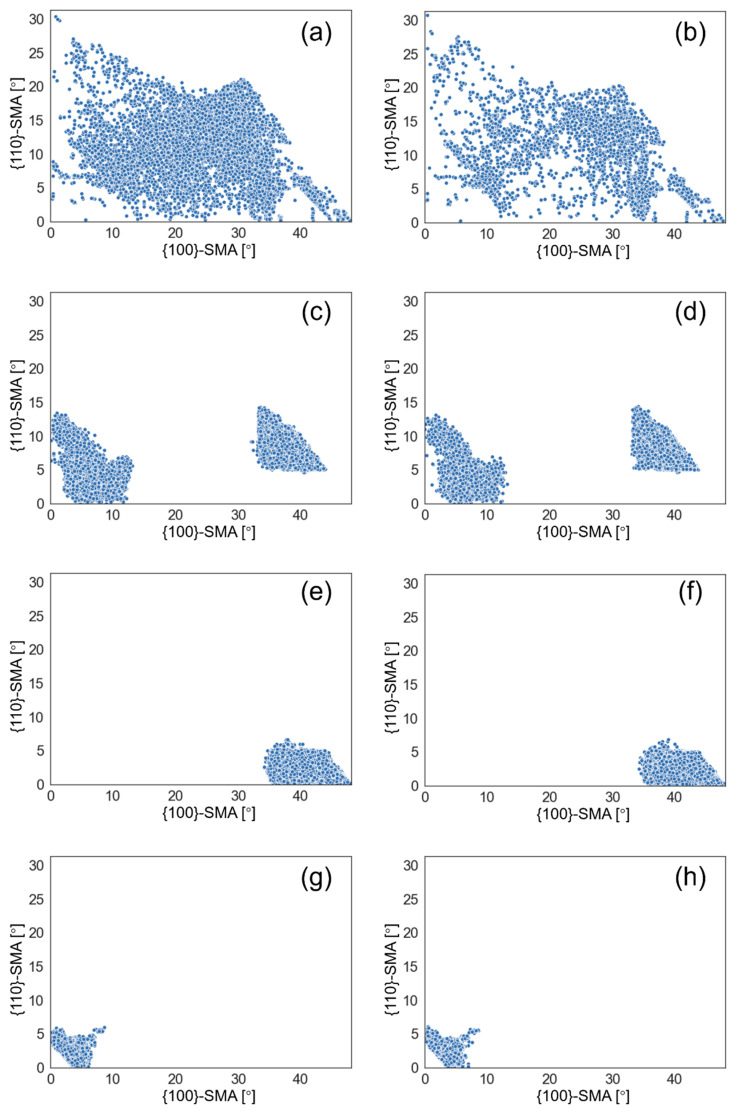
The relationship between {100}-SMA and {110}-SMA of different types of boundaries ((**a**,**b**) PAGBs; (**c**,**d**) packet boundaries; (**e**,**f**) block boundaries; and (**g**,**h**) sub-block boundaries) in: A930 (**a**,**c**,**e**,**g**); and A1050 (**b**,**d**,**f**,**h**).

To a significant extent, the density of each type of boundaries determines the refinement effect of corresponding crystallographic unit. Utilizing pole figures of EBSD measurements, the relationship between variants can be easily determined, and then all the boundaries (θ > 5°) can be classified as PAG boundaries, packet boundaries, block boundaries, and sub-block boundaries, as shown in Figure 9. Figure 10 shows a comparison of {100}-SMAs and {110}-SMAs of different types of boundaries in A930 and A1050 steels. The distribution features of one identical type of boundary for the two samples are very similar, but, for different types of boundaries, the distribution features are quite different. PAG boundaries cover large range on both {100}-SMA and {110}-SMA, and there is no obvious regularity in the distribution, while the other three types of boundaries apparently exhibit clustering. Packet boundaries form two parts or clusters and form a gap between 15° and 30° {100}-SMA, but, for both parts, the {110}-SMAs can be as high as 13°. Block boundaries can only have {100}-SMAs greater than 30° and {110}-SMAs lower than 7°, while sub-block boundaries can only have {100}-SMAs lower than 10° and {110}-SMAs lower than 7°. Thus, theoretically, PAG boundaries and packet boundaries contribute to strength and ductility more than the other two types of boundaries. Block boundaries, part of PAG boundaries and part of packet boundaries refine the microstructure to prevent cleavage fracture. In contrast, the contribution from sub-block boundaries is significantly weaker.

**Figure 11:**
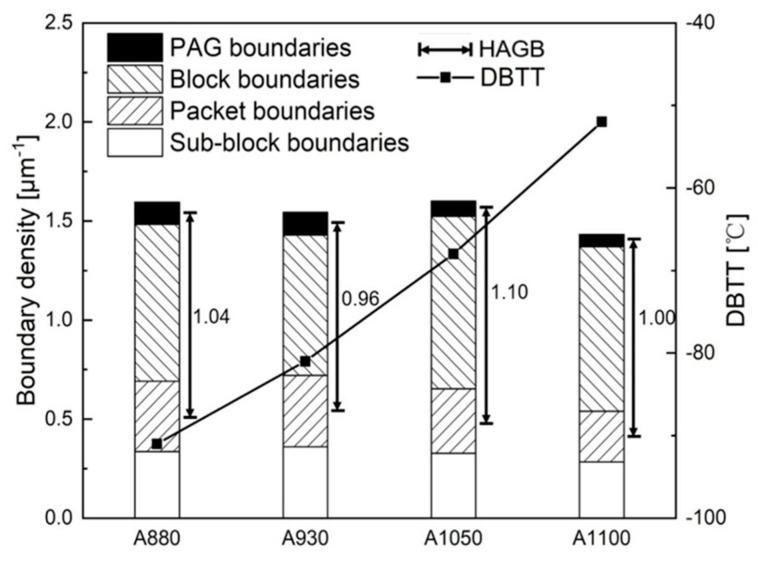
Grain boundaries density of different types and DBTT in experimental steels.

The present study also brings a new perspective to further understand HAGB and its effect on toughness. Apparently, HAGBs, no matter what the criterion is (θ > 15° or θ > 45°), can be partly block boundaries, packet boundaries, or PAG boundaries. All three types of boundaries would have a positive effect on toughening; thus, θ > 15° is a more reasonable criterion to define HAGB rather than θ > 45°, because a large content of PAG boundaries and a small content of packet boundaries have misorientation angles between 15° and 45°. However, sometimes θ > 45° is a good criterion to find boundaries which can effectively inhibit cleavage fracture, because, on using θ > 45°, the packet boundaries with high {100}-SMAs can be distinguished, i.e., the right part of boundaries in Figure 10c,d. These packet boundaries contribute more to toughness because their {100}-SMAs are appreciably greater. The underlying basis of this inference is that specific misorientation angles would never be greater than the corresponding overall misorientation angles. The negative aspect of criterion θ > 45° is that HAGBs may exclude some PAG boundaries whose misorientation angle is less than 45°. However, in some cases, it is not so apparent whether it will influence the effectiveness of analyzed results, which depends on the proportion of PAG boundaries in all boundaries. As shown in Figure 11, in this study, the density of block boundaries was higher than the other three types of boundaries. However, the sample which had the highest density of HAGBs (θ > 45°) and highest density of block boundaries was A1050, but A880 and A930 still had lower DBTT. The most likely reason is that A880 and A930 have higher densities of PAG boundaries and packet boundaries and the differences between the densities of different types of boundaries are insignificant. Several studies have shown that HAGBs with θ > 45° influence toughness, particularly in the weld heat affected zone [18,19,32,33]. In these cases, the austenitization temperatures were always relatively high and large PAGs were obtained, such that block boundaries and packet boundaries dominated in proportion, the contributions from PAG boundaries was limited or similar. Thus, DBTT was determined by the density of high angle packet boundaries and block boundaries to a large extent. In one particular study, it was shown that V1–V2 variant boundary, which is one of the block boundaries, significantly influences toughness [34]. This is absolutely possible and reasonable because it is based on the premise that V1–V2 variant boundary dominates in quantity or the density difference of other types of boundary is small. Meanwhile, the shape of boundaries and the relationship between crack propagation direction [35] also affects the fracture behavior. Moreover, other factors such as density of solid solution atoms, dislocations, precipitates, and texture can also influence toughness and DBTT [28,36,37]. More quantitative characterizations and analyses are needed to reveal the natural rule between mechanical properties and microstructures. To the best of our understanding, the present study is a first attempt to reveal the natural relationship between toughness and microstructure of steels, from the perspective of the different types of boundaries illustrated in Figure 1. A clearer understanding of the characteristics of each type of boundary can help to establish a better relationship between the microstructure and performance.

## 4. Conclusions

Coherent transformation can refine the microstructure of steels and reduce DBTT by improving ductility and inhibiting cleavage fracture; however, not all boundaries contribute equally to toughness. The results of our study indicate that misorientation of specific crystallographic planes provide more detailed information compared to the overall misorientation angle with respect to the enhancement of toughness improvement. The contribution of different types of boundaries on toughness can be summarized as follows:

1. In general, prior austenite grain boundaries have high {100}-plane misorientation angles and high {110}-plane misorientation angles, such that the refinement of prior austenite grains is an effective approach to improve toughness from the perspective of improving ductility and inhibiting cleavage fracture.
2. Packet boundaries can also improve toughness from two perspectives, but only a proportion of packet boundaries can form very low {100}-plane misorientation angles, thus not all packet boundaries are very effective on inhibiting cleavage fracture.
3. Block boundaries are characterized by high {100}-plane misorientation angles and low {110}-plane misorientation angles, and thus their contribution in inhibiting cleavage fracture is more significant than ductility improvement.
4. Sub-block boundaries have low {100}-plane misorientation angles and low {110}-plane misorientation angles, and thus the contribution of sub-block boundaries on toughness improvement is significantly weaker.

## Figures and Tables

**Table 1:** Mechanical properties of experimental steel samples.

Samples	Yield Strength/MPa	Tensile Strength/MPa	Total Elongation/%	DBTT/°C
A880	716	782	23.4	−97
A930	693	756	23.3	−81
A1050	626	741	22.0	−68
A1100	639	727	21.3	−52

**Table 2:** Crystallographic parameters of variants for K-S relationship [6,27].

Variant No.	Misorientation Angle/°	Plane Parallel (CP Group)	Directional Parallel	Bain Group
V1	-	(1 1 1)_fcc_//(0 1 1)_bcc_ (CP1)	[−1 0 1]_fcc_//[−1 −1 1]_bcc_	B1
V2	60.00		[−1 0 1]_fcc_//[−1 1 −1]_bcc_	B2
V3	60.00		[0 1 −1]_fcc_//[−1 −1 1]_bcc_	B3
V4	10.52		[0 1 −1]_fcc_//[−1 1 −1]_bcc_	B1
V5	60.00		[1 −1 0]_fcc_//[−1 −1 1]_bcc_	B2
V6	49.48		[1 −1 0]_fcc_//[−1 1 −1]_bcc_	B3
V7	49.47	(1 −1 1)_fcc_//(0 1 1)_bcc_ (CP2)	[1 0 −1]_fcc_//[−1 −1 1]_bcc_	B2
V8	10.53		[1 0 −1]_fcc_//[−1 1 −1]_bcc_	B1
V9	50.51		[−1 −1 0]_fcc_//[−1 −1 1]_bcc_	B3
V10	50.51		[−1 −1 0]_fcc_//[−1 1 −1]_bcc_	B2
V11	14.88		[0 1 1]_fcc_//[−1 −1 1]_bcc_	B1
V12	57.22		[0 1 1]_fcc_//[−1 1 −1]_bcc_	B3
V13	14.88	(−1 1 1)_fcc_//(0 1 1)_bcc_ (CP3)	[0 −1 1]_fcc_//[−1 −1 1]_bcc_	B1
V14	50.51		[0 −1 1]_fcc_//[−1 1 −1]_bcc_	B3
V15	57.21		[−1 0 −1]_fcc_//[−1 −1 1]_bcc’_	B2
V16	20.60		[−1 0 −1]_fcc_//[−1 1 −1]_bcc_	B1
V17	51.73		[1 1 0]_fcc_//[−1 −1 1]_bcc_	B3
V18	47.12		[1 1 0]_fcc_//[−1 1 −1]_bcc_	B2
V19	50.51	(1 1 −1)_fcc_//(0 1 1)_bcc_ (CP4)	[−1 1 0]_fcc_//[−1 −1 1]_bcc_	B3
V20	57.21		[−1 1 0]_fcc_//[−1 1 −1]_bcc_	B2
V21	20.60		[0 −1 −1]_fcc_//[−1 −1 1]_bcc_	B1
V22	47.12		[0 −1 −1]_fcc_//[−1 1 −1]_bcc_	B3
V23	57.21		[1 0 1]_fcc_//[−1 −1 1]_bcc_	B2
V24	21.05		[1 0 1]_fcc_//[−1 1 −1]_bcc_	B1
